# It Is Not Bad to Be the Big Fish in a Small Pond: Revisiting the Double-Edged Sword Model of College Students’ Perceived Overqualification

**DOI:** 10.3390/bs13070546

**Published:** 2023-06-29

**Authors:** Runsheng Pan, Zhijin Hou, Danni Wang, Beibei Liu

**Affiliations:** 1 Faculty of Psychology, Beijing Normal University, Beijing 100875, China; 202131061048@mail.bnu.edu.cn (R.P.); 202021061100@mail.bnu.edu.cn (B.L.); 2 Normal College, Jimei University, Xiamen 361000, China; bnuwdn@163.com

**Keywords:** perceived overqualification, relative deprivation, achievement goals, learning engagement, life satisfaction, growth mindset

## Abstract

School psychologists are concerned about underperforming students; however, a recent study calls attention to a group of college students who believe themselves to outperform other students: students who perceive themselves as overqualified. In this study, we revisited the double-edged sword model of college students’ perceived overqualification (POQ) by untangling the mediating mechanism between POQ, learning engagement, and life satisfaction. We also tested the interactions between the growth mindset and POQ. Two questionnaire surveys were conducted and attained some different results from previous studies: (1) POQ positively predicted learning engagement via the mediation of career aspiration and performance-approach goals but not performance-avoidance goals; (2) The positive effects of POQ on career aspirations, performance-approach goals, and learning engagement were weakened by the growth mindset; (3) The relationship between POQ and life satisfaction was nonsignificant. Relative deprivation negatively mediated this relationship, while generalized self-efficacy positively mediated this relationship. These findings enriched our understanding of how POQ may affect college students’ learning and well-being; in addition, we also provided initial evidence that a growth mindset is less beneficial for members of advantaged groups in academic settings. Based on our findings, we offered practical suggestions regarding POQ students in colleges.

## 1. Introduction

Perceived overqualification (POQ), known as “being a big fish in a small pond”, has been recognized as an important predictor of employees’ work attitudes and psychological well-being [1]. Since there is evidence suggesting that a similar mismatch between students and colleges also exists [2], some researchers anticipated that college students may also perceive themselves to be overqualified to attend their colleges [3]. Although most extant studies regard POQ as an unfavorable condition [4,5], they recognize POQ as a double-edged sword for college newcomers: POQ negatively predicted college students’ life satisfaction while positively predicting learning motivation at the same time [3].

In this study, we revisited this model for three primary reasons. First, this model, especially the positive relationship between POQ and learning motivation, requires replicated validation when considering the large number of studies that report negative consequences of POQ in the workplace [4] and undermatch in colleges [6,7]. Second, one shortcoming of the aforementioned study is that their participants were all freshmen who just entered college; meanwhile, there is evidence suggesting that POQ is not stable for newcomers [3,8]. Therefore, it is necessary to revisit the model with college seniors. Third, based on their findings, Wang et al. [3] put forward a dual-motivation model between POQ and learning engagement, which requires further validation. Addressing this dual-motivation model, we introduced career aspirations and performance-approach goals as parallel mediators between POQ and learning engagement. In the following section, we shall elaborate on our theoretical understanding of the model and then propose our hypothesis. Because studies on college students’ POQ are extremely rare, we mainly reviewed POQ studies in the workplace. Another reason for doing so is that we found POQ studies to be more theoretically driven compared with undermatch studies.

Another question that we are interested in is the boundary condition of the growth mindset. Although the benefits of a growth mindset have already been widely recognized [9,10], researchers have also challenged whether its positive effect applies to students who already feel confident about their abilities [11]. POQ students meet this criterion perfectly. Therefore, we aimed to provide more evidence on this topic by investigating the interactions between POQ and growth mindset.

The current study contributes to the existing literature in several ways. First, this study is one of the few that addresses the subjective experience of college students who believe themselves to be under matched. Doing so helps researchers better understand how undermatch affects college students’ academic performance and mental well-being. Second, this study is one of the few that focuses on the POQ of college students instead of employees. Studying the same variable with different groups helps researchers better understand the nature of the variable. Third, this study contributes to mindset theory by exploring its boundary conditions in academic settings. Finally, college counselors can apply our findings to their practice when they have a student client who believes themselves to be overqualified or undermatched.

### 1.1. Perceived Overqualification among College Students

POQ of college students refers to “college students who believe their actual qualifications exceed the requirements of being admitted to the current college and worry that their potential can’t be fully developed in this college” [3]. The concept of POQ, originally developed in the field of organizational psychology, describes the situation in which employees believe their KSAs (knowledge, skills, and abilities) exceed the job requirements and that they are not being fully utilized in their actual work [12]. In higher education practice, a similar mismatch between colleges and students observed: undermatch occurs when students cannot attend selective universities that match their academic potential [6,7]. To our knowledge, most POQ and undermatch studies have revealed negative consequences for employees and college students. For example, POQ negatively predicts employees’ job satisfaction [13], mental health [12], and adjustment [8]. Regarding college students, undermatch is associated with low satisfaction on campus [5], a low rate of bachelor’s degree completion [14], and unfavorable post-college outcomes [2].

In existing undermatch studies, researchers tend to use various objective indicators to determine who is undermatched. The perfect match approach, one of the most commonly used methods, uses students’ possibility of attending selective universities predicted by a set of students’ previous academic achievements (such as SAT scores, high school GPA, and high school math grades) as an indicator of undermatch [15]. In contrast, in extant POQ studies, researchers prefer self-reported measurements [4]. Though an objective measure is less affected by common method bias, organizational psychologists are more interested in subjective overqualification because they believe that subjective overqualification better captures the subtle individual differences that cannot be fully reflected by objective indicators [8]. For example, two employees with similar educational backgrounds may perform completely differently in the same position. Although subjective overqualification and objective overqualification showed some convergence (*r* = 0.40) [1], Khan and Morrow [16] found that subjective overqualification had better predictive power than objective overqualification on psychological well-being.

Given the above reasons, we are more interested in the subjective experience of college students. To the best of our knowledge, only two existing studies focus on POQ in college learning. Interestingly, those two studies obtained contradictory results: one study found that POQ negatively predicted learning interest and learning input [17], while the other one found that POQ positively predicted learning motivation [3]. Although the results of the first study are consistent with most extant studies that demonstrate the negative consequences of POQ, we are more in favor of the latter because their POQ measurement is more reliable. To the best of our knowledge, there was no published reliable measurement tool of college students’ POQ until their study. They developed their questionnaire with a thematic analysis approach and reported acceptable psychometric properties, including internal consistency, test-retest reliability, convergent and discrimination validity, and structural validity. In contrast, Xu et al. [17] did not specify their POQ measurement and only reported internal consistency rather than other psychometric properties.

### 1.2. Perceived Overqualification as a Motivator for Learning Engagement

To explain the outcomes of POQ, relative deprivation theory is one of the most commonly referenced theories [4]. Drawing on relative deprivation theory, POQ is recognized as a demotivator because it represents a sense of being deprived of deserved jobs [4]. This explains why POQ is always associated with negative consequences, especially job attitudes [1]. Although Sicherman and Galor [18] believed that overeducated employees would move to more qualified positions in a short time, this hypothesis is hardly supported by empirical studies; in contrast, most studies report that POQ will lead to turnover intention and turnover behavior [1,4].

However, in the framework of relative deprivation theory, we would like to take a more positive view of the relationship between POQ and turnover intention. Theoretically, being relatively deprived individually does not always lead to negative outcomes [19]. This is because relative deprivation motivates people to take actions to improve their situations: sometimes in destructive ways, such as crimes [20], sometimes in avoidant ways, such as drug abuse [21], or sometimes through achievement, such as academic engagement [22]. Cognitive psychologists also suggested potential positive outcomes from individual relative deprivation. Zhang et al. [23] found that participants’ working memory capacity increased after inducing individual relative deprivation. For employers and managers, turnover has long been regarded as a reflection of job dissatisfaction and a signal of employee loss [24,25]. Likewise, extant studies focus extensively on POQ’s effect on variables that affect organizations directly or indirectly (e.g., job attitudes, counterproductive behaviors). Only a few studies care about POQ employees’ personal career development. From POQ employees’ perspective, leaving a dissatisfying position could be the first step in career advancement, which gives them a second chance to attain a more favorable job. Indeed, Maynard and Parfyonova [26] found that POQ not only predicted turnover intention but also predicted new job attainments six months later; moreover, those individuals reported lower POQ in their new positions. The evidence above implies that such motivation is carried by the POQ employees.

Given the theoretical foundation of relative deprivation theory and the empirical evidence of the relationship between POQ and turnover, it is plausible to predict that POQ motivates individuals to move to more satisfying positions. Ideally, POQ college students should be motivated to attend a more selective university. However, college students are not able to change colleges as easily as employees can change jobs. For example, in China, perhaps the only way is to drop out of the current college and repeat another year of high school for a second chance to take the college entrance examination, which costs a considerable amount of time and money but does not guarantee success, let alone the mental pressure. On the bright side, compared with overqualified jobs, colleges offer more opportunities for personal growth: students can always learn knowledge and build skills regardless of the selectivity of the college, though such opportunities could be enhanced at more competitive universities. After graduation, postgraduate and employment opportunities that match their qualifications would reward those talented and hard-working students. In contrast, POQ employees often feel there are no opportunities for career advancement in their positions [12]. In summary, POQ employees enjoy fewer internal opportunities but more external opportunities, which leads to spontaneous turnover. In contrast, POQ students enjoy fewer external opportunities but more internal opportunities, which should lead to more efforts into academic activities that contribute to their future success.

In addition to relative deprivation theory, Wang et al. [3] proposed their hypothesis from another perspective—cognitive consistency theory. They suggested that POQ students are likely to experience cognitive inconsistency: on the one hand, they believe they are qualified for a more selective university, yet on the other hand, they have to study at the current institution. According to cognitive consistency theory, people are motivated to eliminate such inconsistencies between their cognitions and behavior [27]. Although people can reach consistency by various means, such as admitting to not being overqualified, the perception of overqualification is bounded by one’s core self-evaluation. For college students, this cognitive inconsistency could also be magnified by the social norm of judging people’s ability and intelligence by the university they attend; as a result, it also creates inconsistency between self-evaluation and social recognition. Therefore, we believe that POQ students should be motivated to demonstrate their overqualifications in college learning; in this way, they can regain cognitive consistency and maintain a positive self-evaluation. Likely, other researchers also posit that POQ activates employees’ goal of maintaining positive self-evaluation [28].

### 1.3. The Dual-Motivation Hypothesis of Perceived Overqualification

It seems that both relative deprivation theory and cognitive consistency theory suggest that POQ college students are motivated to engage in learning activities. Consistent with this hypothesis, Wang et al. [3] found that POQ positively predicted learning motivation. However, in reviewing their findings, they hypothesized that those two theoretical orientations may reflect two different facets of POQ’s motivation. Specifically, relative deprivation theory implies that POQ individuals are motivated to pursue something external (i.e., qualified colleges or jobs); in contrast, cognitive consistency theory implies that POQ individuals are motivated to pursue something internal (i.e., to confirm and maintain a positive self-image).

Since this dual-motivation hypothesis has yet to be verified, operationalizing and verifying this dual-motivation hypothesis is one of the goals of this study. Within the framework of relative deprivation theory, we operationalized college students’ desire to improve their situation as a career aspiration. Career aspiration refers to the education, achievement, and leadership achievements that people desire to accomplish [29], and it serves as a motivator for career advancement [30]. Researchers have found that high career aspiration usually predicts academic achievements and career success [31,32]. As discussed above, most POQ students may not be able to improve their situations by changing colleges directly; instead, a more realistic approach is to aim for better postgraduate or employment opportunities after graduation. As a result, POQ students should possess higher aspirations for their future employment or education than students who feel just qualified. Therefore, we proposed that POQ positively predicts career aspiration (Hypothesis 1).

Within the framework of cognitive consistency theory, we operationalized POQ students’ desire to prove their overqualifications as performance-approach goals. According to the trichotomous achievement goals theory [33], students are usually motivated by three different achievement goals, including a master goal (a self-referenced goal that focuses on ability enhancement and knowledge gain), a performance-approach goal (an other-referenced goal that focuses on demonstrating competence relative to others), and a performance-avoidance goal (an other-referenced goal that focuses on avoiding incompetence relative to others). According to the definitions, the desire to prove overqualification is a type of performance-approach goal: it requires POQ students to demonstrate competence relative to other students. For discrimination validity, we also include performance-avoidance goals in our model: we believe performance-avoidance goals are far from sufficient for POQ students because avoiding incompetence is not enough to prove their overqualifications. Accordingly, we proposed that POQ positively predicts performance-approach goals (Hypothesis 2) and that POQ does not predict performance-avoidance goals (Hypothesis 3).

Since Xu et al. [17] reported that POQ negatively predicted learning input, we want to further investigate whether the motivations of POQ are truly related to engagement in learning activities. Learning engagement is so important that it has been recognized as the “holy grail” of learning [34]. As important motivators, both career aspiration and performance-approach goals were found to be associated with positive academic outcomes [35,36,37]. Therefore, we believe the career aspiration and performance-approach goals evoked by POQ would lead to positive engagement in learning activities. Accordingly, we proposed that POQ positively predicts learning engagement via the mediation of career aspiration (Hypothesis 4) and the mediation of performance-approach goals (Hypothesis 5).

### 1.4. When a Growth Mindset Is Less Beneficial

In the past decade, growth mindset has been recognized as an important predictor of academic progress [38]. Mindset is defined as “the core assumptions about the malleability of personal qualities” [10]. One’s mindset usually falls on a continuum between a fixed mindset and a growth mindset. A person with a fixed mindset usually believes personal attributes such as intelligence are stable and barely change, while a person with a growth mindset usually believes they are malleable and can be changed by effort and experience [39]. With a growth mindset, failure is viewed as a result of a lack of effort instead of a lack of competence, which is beneficial for pursuing long-term goals that require persistent striving.

Although educators have already started to apply mindset theory in their practice [40], a recent meta-analysis suggested that the relationship between growth mindset and academic achievements (*r* = 0.10), as well as the effect of growth mindset intervention on performance improvement (*d* = 0.08), is rather weak [11]. The authors offered a hypothesis to explain this result: a growth mindset may be less beneficial or even detrimental for students who are already confident about their abilities because a growth mindset may make their advantages fragile. Studies of stereotype threat provided initial evidence: Mendoza-Denton et al. [41] found that a fixed mindset can boost the performance of favorable stereotyped groups: when math stereotypes were made salient (Asian students vs. white students; males vs. females), both Asian students and male students performed better under the fixed mindset condition than under the growth mindset condition.

According to the qualitative research conducted by Wang et al. [3], POQ students are characterized by being confident about their abilities. They believe that their abilities are superior to those of the average student in their colleges. Therefore, we can anticipate a similar effect of having a growth mindset on POQ students. Accordingly, we proposed that a growth mindset weakens the positive relationship between POQ and career aspiration (Hypothesis 6) and performance-approach goals (Hypothesis 7).

### 1.5. Perceived Overqualification as a Risk Factor for Life Satisfaction

Unlike the relationship between POQ and learning motivation, the relationship between POQ and life satisfaction was well supported by both relative deprivation theory and empirical studies in the workplace [1,4], as well as undermatch studies in colleges [5]. As highlighted by relative deprivation theory, being relatively deprived leads to negative emotions such as anger and dissatisfaction [42]. Since POQ represents a sense of being deprived of working/learning opportunities that match one’s qualifications, it is reasonable to predict that POQ individuals will have low levels of life satisfaction. Empirical evidence also supported the negative relationship between POQ and life satisfaction, regardless of whether they were employees [43,44] or college students [3].

Despite the theoretical argument, researchers rarely tested relative deprivation as a mediator in the relationship between POQ and life satisfaction [44]. Hence, we aimed to test the nomological network of such relationships in the framework of relative deprivation theory. Accordingly, we proposed that POQ positively predicts relative deprivation (Hypothesis 8) and negatively predicts life satisfaction (Hypothesis 9); and that POQ negatively predicts life satisfaction via the mediation of relative deprivation (Hypothesis 10).

**Figure 1:**
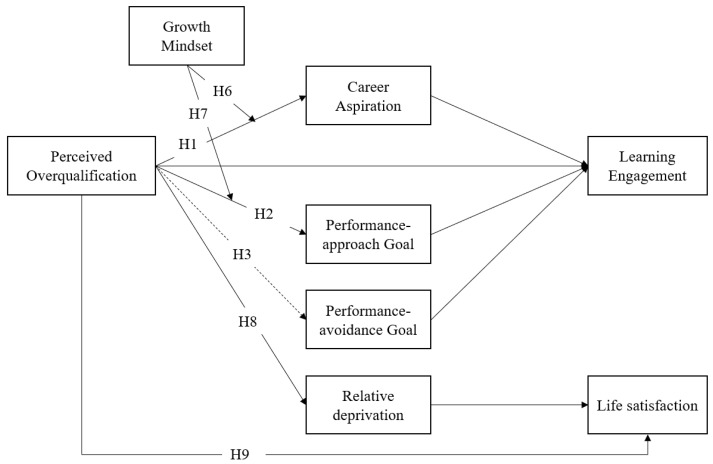
Hypothesized relationships between perceived overqualification, learning engagement, and life satisfaction. H4: POQ—Career aspiration—Learning engagement; H5: POQ—Performance-approach goal—Learning engagement; H10: POQ—Relative deprivation—Life satisfaction.

In summary, our hypothesized model is illustrated in Figure 1.

**Study 1**

## 2. Materials and Methods

### 2.1. Participants

Participants were volunteer students recruited from four colleges in Gansu Province, China. The survey was a part of the college student psychological well-being survey, and it received 894 initial responses. Three quality check items (e.g., Please select strongly disagree for this item) were adopted to rule out invalid responses, and responses that failed on more than one quality check item were dropped. As a result, 663 valid responses were received. Among these responses, 407 (61.39%) identified themselves as female, 254 (38.31%) identified themselves as male, and 2 participants did not report their gender. The average age was 19.92 years. Regarding grade, 258 (38.9%) were sophomores, 228 (34.4%) were juniors, 149 (22.5%) were seniors, and 28 did not report their grades. We did not recruit freshmen because we planned to test the hypothesized model with students who already had some experience in college. Participants also varied in majors: 339 (51.1%) were STEM majors, 179 (27.0%) were business majors, 66 (10.0%) were medical students, 70 (10.6%) were art majors, and 9 did not specify their majors. This study was carried out in accordance with the recommendations of the World Medical Association’s Declaration of Helsinki. Informed consents were collected before the survey. Participants were informed of their confidentiality and freedom to withdraw at any time. After completing the questionnaire, participants were debriefed and received 5 RMB yuan for compensation.

### 2.2. Measurements

The Chinese college student perceived overqualification questionnaire [3] was adopted in this study. This questionnaire contains 20 items. Participants were asked to respond on a 5-point Likert scale. We calculated the mean score of each subscale, and the mean score of the subscale score is calculated to reflect POQ. A higher score indicates a higher POQ. A sample item is “Those who are not as good as I am can also be admitted to my current college/major”. The detailed psychometric properties of the questionnaire are reported in a later section.

The achievement aspiration subscale and educational aspiration subscale from The career aspiration scale-revised [30] were adopted in this study. We chose those two subscales because we believe they better capture the motivation enabled by POQ (the motivation towards better employment or postgraduate opportunities) in comparison to leadership aspiration. The original scale contains 23 items. Participants were asked to respond on a 5-point Likert scale. We calculated the mean score of each subscale, and a higher score indicated higher aspirations. A sample item for achievement aspiration is “I want to be among the very best in my field”. In this study, the Cronbach’s alpha coefficients of the achievement aspiration subscale and educational aspiration subscale were 0.81 and 0.89, respectively.

The performance-approach goal subscale and performance-avoidance goal subscales from The Chinese college students achievement goal orientation questionnaire [45] were adopted in this study. This Chinese version was revised from the original trichotomous achievement goal scale [33]. This questionnaire contains 13 items. Participants were asked to respond on a 5-point Likert scale. The mean score of each subscale was calculated. A sample item is “It is important for me to do better than other students” (performance-approach goals). In this study, the Cronbach’s alpha coefficients of the two adopted subscales were 0.85 and 0.67.

The Chinese college students learning engagement scale [46] was adopted in this study. This scale contains 18 items. Participants were asked to respond on a 5-point Likert scale. We calculated the mean score, and a higher score indicates greater learning engagement. A sample item is “I always listen carefully and think actively in class”. In this study, the Cronbach’s alpha coefficient was 0.93.

The growth mindset scale [39] was adopted in this study. This scale contains 20 items. Participants were asked to respond on a 4-point scale. The scale is unidimensional, and we calculated the mean score, and a higher score indicated a higher growth mindset. A sample item is “To be honest, you can’t really change how smart you are” (reversed scoring). In this study, the Cronbach’s alpha coefficient was 0.77.

The relative deprivation scale [47] was adopted in this study. This scale contains four items, and we calculated the mean score. Participants were asked to respond on a 6-point scale. A sample item is “Considering the efforts I have made, my life should have been better”. In this study, the Cronbach’s alpha coefficient was 0.74.

The life satisfaction questionnaire [48] was adopted in this study. The questionnaire contains five items, and we calculated the mean score. Participants were asked to respond on a 7-point scale. A sample item is “I am satisfied with my life”. In this study, the Cronbach’s alpha coefficient was 0.84.

## 3. Results

Descriptive analysis was handled with SPSS 26.0; confirmatory factor analysis was handled with Mplus 8.3; the measurement invariance test was carried out with the semTools 0.5–6 package [49] in the R 4.2.1 environment, and path analysis was handled with Process 3.5 [50] in SPSS 26.0.

### 3.1. Common Method Bias

Common method bias was examined with Harmon’s single-factor method. Exploratory factor analysis suggested that 19.22% of the total variance was explained by the first factor, which is lower than the 50% rule of thumb [51]. The results indicated that common-method bias did not threaten the validity of the study.

### 3.2. Psychometric Properties of Chinese College Students’ Perceived Overqualification Questionnaire

Because this study enrolled college students from different grades, it is necessary to ensure that the POQ questionnaire has the same meaning regardless of their college years. Therefore, we conducted measurement invariance tests across grades. In addition, we also tested the internal consistency and structural validity of the questionnaire.

We applied Cronbach’s alpha coefficient as an indicator of internal consistency. The results suggested an overall good internal consistency: the whole questionnaire scored 0.89, and the 4 subscales scored 0.78, 0.90, 0.78, and 0.87, respectively.

We conducted a confirmatory factor analysis to evaluate structural validity. The goodness-of-fit indices indicated an overall good structural validity: χ^2^/df = 4.32, CFI = 0.92, TLI = 0.90, RMSEA = 0.07, SRMR = 0.06. Except for the RMSEA being slightly over the criteria suggested by Hu and Bentler [52] (χ^2^/df < 5, CFI > 0.90, TLI > 0.90, RMSEA < 0.06, SRMR < 0.08), the results met the acceptable standard for confirmatory factor analysis. 

Following the guidance provided by Putnick and Bornstein [53], we conducted a step-by-step strict measurement invariance test in the structural equation modeling framework. Specifically, the model fits four models, including the configural invariance model, metric invariance model, scalar invariance model, and residual invariance model, were compared step by step, and a nonsignificant model difference indicates measurement invariance. As presented in Table 1, the configural model demonstrated an acceptable model fit, and was used as the baseline model in the following comparison. Because the sensitivity of the Chi-square test may be affected by our relatively large sample size (*n* > 500), we applied changes in CFI and RMSEA, the alternative fit indices, as criteria for model comparison. According to Chen [54], a change in CFI less than 0.01 and a change in RMSEA less than 0.015 indicate measurement invariance. The comparison results (ΔCFI < 0.002 and ΔRMSEA < 0.002) indicated that there were no significant differences between the compared models for each step. Therefore, the Chinese college student’s perceived overqualification questionnaire has the same meaning for participants in this study regardless of their grades.

### 3.3. Descriptive Analysis

Descriptive results are presented in Table 2. Participants scored medium-high on all variables except relative deprivation (M = 2.58). POQ was positively correlated with achievement aspiration (*r* = 0.22, *p* < 0.01), educational aspiration (*r* = 0.25, *p* < 0.01), performance-approach goal (*r* = 0.29, *p* < 0.01), learning engagement (*r* = 0.29, *p* < 0.01), and relative deprivation (*r* = 0.24, *p* < 0.01).

### 3.4. Testing Perceived Overqualification as a Motivator for Learning Engagement

We first tested the hypothesis of POQ as a positive motivator of learning engagement. Mediation analysis was conducted with model 4 in Process 3.5. Participants’ gender, grade, and family monthly income were set as control variables. Because the correlation between achievement aspiration and educational aspiration was relatively high (*r* = 0.60), we merged them into one variable—career aspiration—by calculating the mean to simplify the model and avoid multicollinearity (We also analyzed achievement aspiration and educational aspiration as independent mediators in one model, and the results suggested that the mediating effect of achievement aspiration was no longer significant (the mediating effect of educational aspiration was still significant); however, when they were analyzed separately, both achievement aspiration and educational aspiration showed significant mediating effects). The results of the direct effect analysis are presented in Table 3. The POQ’s direct effect on career aspiration (*β* = 0.25, *p* < 0.01), performance-approach goals (*β* = 0.41, *p* < 0.01), and learning engagement (*β* = 0.32, *p* < 0.01) were significant. As expected, POQ did not predict performance-avoidance goals (*β* = 0.02, *p* = 0.76). When mediators entered the regression model, R2 improved from 0.10 to 0.26. In the mediating model, POQ’s direct effect on learning engagement is still significant (*β* = 0.17, *p* < 0.01); in addition, career aspirations (*β* = 0.38, *p* < 0.01) and performance-approach goals (*β* = 0.13, *p* < 0.01) positively predicted learning engagement. Therefore, Hypotheses 1, 2, and 3 were supported by the data. The relationship between POQ and learning engagement was partially mediated by career aspiration and performance-approach goals.

Results from bootstrap analysis with 5000 resamples further revealed the hypothesized mediating effects. As presented in Table 4, POQ’s total effect (effect = 0.32, 95% CI: [0.25, 0.40]) and direct effect (effect = 0.17, 95% CI: [0.10, 0.25]) on learning engagement were significant; in addition, the indirect effect via career aspiration (effect = 0.10, 95% CI: [0.06, 0.15]) and performance-approach goals (effect = 0.05, 95% CI: [0.02, 0.09]) were also significant. In contrast, the indirect effect via performance-avoidance goals was nonsignificant (effect < −0.00, 95% CI: [−0.01, 0.01]). Therefore, Hypotheses 4 and 5 were supported by the data.

### 3.5. Testing Growth Mindset as a Moderator

**Figure 2:**
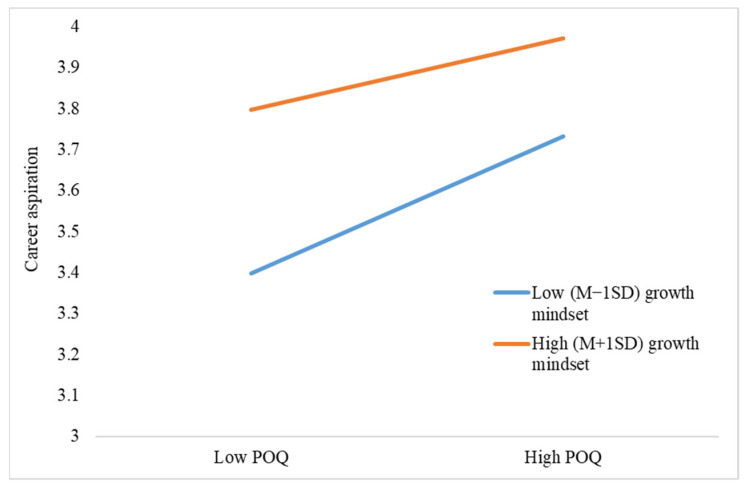
The effect of POQ × growth mindset on career aspiration.

**Figure 3:**
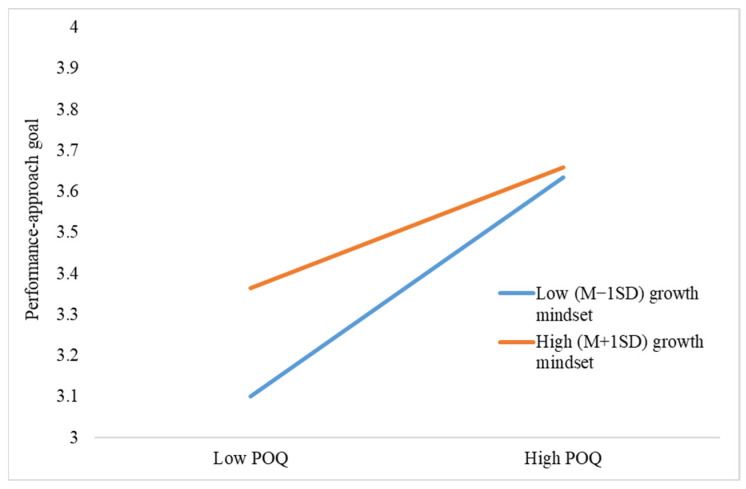
The effect of POQ × growth mindset on performance-approach goal.

**Figure 4:**
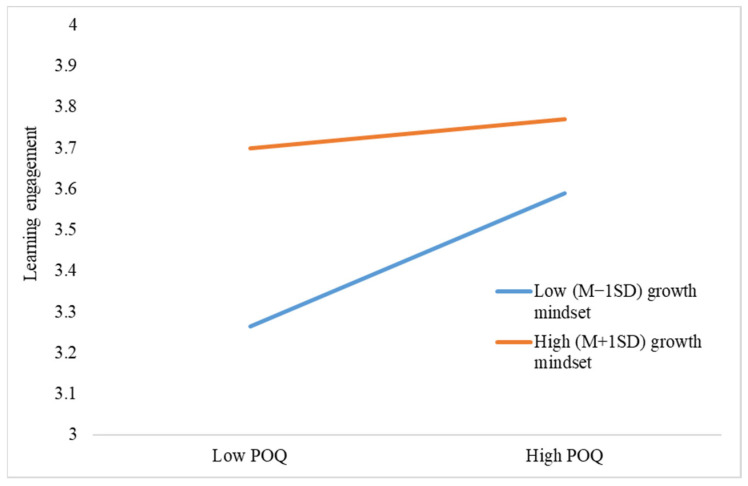
The effect of POQ × growth mindset on learning engagement.

After verifying the mediating model, we then tested the hypothesized moderating effect of growth mindset with model 8 in Process 3.5. Since the direct and indirect effects of performance-avoidance goals were nonsignificant, they were not included in the moderated mediation analysis. The results of the moderating effect analysis are presented in Table 5. The interactions between POQ and growth mindset negatively predicted career aspiration (*β* = −0.32, *p* = 0.01), performance-approach goal (*β* = −0.48, *p* < 0.01), and learning engagement (*β* = −0.52, *p* < 0.01). Therefore, Hypothesis 6 and 7 were supported by the data. The simple-slope style interactions were illustrated in Figure 2, Figure 3 and Figure 4. As reflected by the figures, the positive relationships between POQ and outcomes were stronger for students with a low-growth mindset.

A bootstrap analysis with 5000 resamples was conducted to test the hypothesized moderated mediation effects. As presented in Table 6, POQ’s direct effect on learning engagement is significant for participants with a low growth mindset (effect = 0.32, 95% CI: [0.23, 0.41]); however, it is nonsignificant for participants with a high growth mindset. For the indirect effects via career aspiration and performance-approach goals, both were significant regardless of the level of growth mindset, although the effect was larger for participants with a lower growth mindset.

### 3.6. Testing Perceived Overqualification as a Risk Factor for Life Satisfaction

We then tested the hypothesis of POQ as a risk factor for life satisfaction. Mediation analysis was conducted with model 4 in Process 3.5. Participants’ gender, grade, and family monthly income were set as controlled variables. The results were presented in Table 7. POQ positively predicted relative deprivation (*β* = 0.34, *p* < 0.01) but not life satisfaction (*β* = 0.11, *p* = 0.13). After relative deprivation entered the regression model, POQ’s effect on life satisfaction became significant (*β* = 0.17, *p* = 0.03). Therefore, the data supported Hypothesis 8 but not Hypothesis 9.

A bootstrap analysis with 5000 resamples was conducted to calculate the confidence intervals of the mediating effect. Unexpectedly, the results revealed a nonsignificant total effect (95% CI = [−0.03, 0.26]) and a positive direct effect (95% CI = [0.02, 0.32]) of POQ on life satisfaction. The indirect effect via relative deprivation was negative, as expected (95% CI = [−0.10, −0.02]). Therefore, Hypothesis 10 was supported by the data. To further clarify this issue, we conducted another questionnaire survey in Study 2.

**Figure 5:**
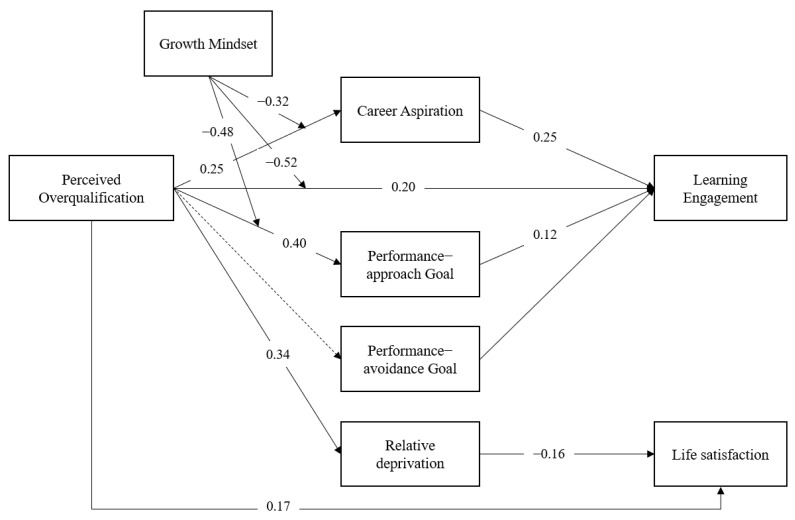
Results of Study 1 (Based on results of moderated mediation model).

In summary, the results of Study 1 (moderated mediation model) can be illustrated in Figure 5.

**Study 2**

The purpose of Study 2 is to replicate and untangle the unexpected relationship between POQ and life satisfaction found in Study 1. In Study 1, although the mediating effect via relative deprivation was negative as expected, the nonsignificant total effect and the positive direct effect of POQ on life satisfaction implied that we missed important mediator(s) that play a reversed role in this relationship.

Theoretically, it is tempting to assume that POQ contributes to life satisfaction via positive self-efficacy. As stated by self-determination theory [55], competence is one of three basic psychological needs, and satisfaction of these needs contributes to both physical and psychological well-being. Consistent with this theory, empirical studies have demonstrated a strong relationship between generalized self-efficacy and life satisfaction [56,57,58]. Generalized self-efficacy refers to a general belief in one’s ability to handle important life events [59]. Since POQ represents a kind of positive evaluation in regard to one’s KSAs [28], employees can likely build positive self-efficacy in the workplace, as long as their surplus KSAs can help them better complete their daily work. However, the positive relationship between POQ and self-efficacy was hardly supported by studies in organizational psychology, with a meta-analysis revealing a weak relationship between POQ and self-efficacy [60].

As an exception, a significant relationship between POQ and role breadth self-efficacy was observed [61,62]. Role breadth self-efficacy refers to confidence in performing tasks outside of prescribed job requirements [63]. Why does POQ predict role-breadth self-efficacy but not generalized self-efficacy? As suggested by Liu and Mo [28], KSA utilization is an important source of self-esteem in the workplace. According to the definition of POQ, the surplus qualifications possessed by POQ employees are hardly utilized in their daily work [64]. A meta-analysis also revealed a nonsignificant relationship between POQ and job performance [1]. Because their surplus qualifications are not utilized, recognized, or rewarded by the organization, their surplus qualifications hardly contribute to their feeling of competence. As found by Liu et al. [65], POQ negatively predicted organization-based self-esteem. However, those surplus qualifications could help cope with accidental demands outside of the formal work tasks; consequently, role-breadth self-efficacy can be built from those practices [61,62].

The patterns between POQ, generalized self-efficacy, and role breadth self-efficacy imply that POQ individuals can build positive self-efficacy as long as the circumstances allow them to exercise their surplus qualifications. In our case, college students feel overqualified because of their surplus learning abilities and academic potential rather than unusable KSAs [3]. Their learning abilities and academic potential demonstrated in high school can still be utilized in college. Moreover, they enjoy a less competitive learning environment in colleges in which they are overqualified, which provides them with more opportunities to be recognized, make achievements, and win competitions. As a result, POQ college students should be able to build their self-efficacy consistently through college learning. Hence, we proposed that POQ positively predicts generalized self-efficacy (Hypothesis 11) and that POQ positively predicts life satisfaction via the mediation of generalized self-efficacy (Hypothesis 12).

## 4. Materials and Methods

### 4.1. Participants

Participants were volunteer students recruited from a college of education (which caused an imbalanced gender ratio) in Gansu Province, China. The college, participants, and experimenters were all different from those in Study 1. The survey received 166 initial responses. Quality check items similar to those in Study 1 were adopted. As a result, 124 valid responses were retained for analysis. Among these responses, 100 (80.65%) identified as female and 24 (19.35%) identified as male. The average age was 19.65 years. Regarding grade, 71 (57.26%) were sophomores, 35 (28.23%) were juniors, and 18 (14.52%) were seniors. We conducted this study in accordance with the recommendations of the World Medical Association’s Declaration of Helsinki. Before the survey, all participants signed informed consents. Participants were informed of the confidentiality of their data and their freedom to withdraw at any time. After the survey, each participant received 5 RMB in compensation.

### 4.2. Measurements

The measurements adopted to measure POQ, relative deprivation, and life satisfaction are the same as those in Study 1. The self-efficacy subscale from The Positive psychological capitals questionnaire [66] was adopted to measure generalized self-efficacy. The self-efficacy subscale consists of seven items. A sample item is “I am very confident about my abilities”. Participants were asked to respond to a 7-point scale. The internal consistency of each measurement was reported along with descriptive results.

## 5. Results

### 5.1. Common Method Bias

Common method bias was examined with Harmon’s single-factor method. Exploratory factor analysis suggested that 26.82% of the total variance was explained by the first factor, which is lower than the 50% rule of thumb [51]. The results indicated that common-method bias did not threaten the validity of the study.

### 5.2. Descriptive Analysis

Descriptive results are presented in Table 8. The correlational analysis demonstrated that generalized self-efficacy positively correlated with POQ (*r* = 0.33, *p* < 0.01) and life satisfaction (*r* = 0.38, *p* < 0.01).

### 5.3. Testing Generalized Self-Efficacy as a Mediator

Mediation analysis was conducted with model 4 in Process 3.5. Gender and grade were set as control variables. In addition, subjective socioeconomic status (SSES), measured by the classic social ladder item (“Imagine this ladder represents Chinese society and the upper bracket indicates higher social status, please indicate your social status on this ladder”), was controlled as another covariate. The results of the direct effect analysis are presented in Table 9. POQ positively predicted relative deprivation (*β* = 0.49, *p* < 0.01), generalized self-efficacy (*β* = 0.47, *p* < 0.01), and life satisfaction (*β* = 0.35, *p* = 0.04). After those two mediators entered the regression model, POQ’s direct effect on life satisfaction was no longer significant (*β* = 0.29, *p* = 0.11). Both relative deprivation and generalized self-efficacy significantly predicted life satisfaction. Replicating the findings in Study 1, the data supported Hypothesis 8 but not Hypothesis 9; in addition, the data supported Hypothesis 11.

A bootstrap analysis with 5000 resamples was conducted to calculate the confidence intervals of mediating effects. The results were presented in Table 10. Replicating the findings in Study 1, the results revealed a nonsignificant total effect (95% CI = [−0.05, 0.58]) of POQ on life satisfaction as well as a negative indirect effect via relative deprivation (95% CI = [−0.34, −0.03]). However, the direct effect was no longer significant when considering the indirect effect via generalized self-efficacy (95% CI = [−0.10, 0.59]), suggesting that introducing generalized self-efficacy as a mediator explained the relationship between POQ and life satisfaction well. As expected, the indirect effect via general self-efficacy was positive (95% CI = [0.04, 0.40]). Therefore, Hypothesis 12 was supported by the data.

## 6. Discussion

Although previous studies highlighted the negative effect of “being a big fish in a small pond”, this study revealed a somewhat positive side of POQ among college seniors. In this study, we verified and extended the double-edged sword model of college students’ POQ [3]. Specifically, we found that college seniors who perceived themselves to be overqualified were characterized by high career aspiration, high performance-approach goals, high learning engagement, and high generalized self-efficacy. In the relationship between POQ and life satisfaction, the positive effect of generalized self-efficacy is strong enough to compensate for the negative effect caused by relative deprivation. In addition, this study provided evidence that a growth mindset is less beneficial for individuals who are already confident about their abilities. We believe those findings have both theoretical and practical implications.

### 6.1. Theoretical Implications

#### 6.1.1. POQ as a Motivator of College Learning

Before this study, limited research yielded contradictory results on the relationship between POQ and learning motivation [3,17]. Carrying on the work done by Wang et al. [3], this study contributed to the field by untangling how POQ motivates college students to engage in college learning. Although POQ as a positive motivator has been neglected in previous studies, the dual-motivation hypothesis of POQ is well supported by two classic social psychology theories: relative deprivation theory [42] and cognitive consistency theory [27].

First, this study contributes to the extant literature by demonstrating POQ as a positive motivator in the framework of relative deprivation theory. According to the review by Smith et al. [19], being relative deprived individually does not always lead to negative consequences. POQ can motivate people to improve their situation and to acquire “what I deserve”. This motivation was not only confirmed by the relationship between POQ and turnover intentions [1,26] but also supported by the relationship between individual relative deprivation and achievement behaviors [19]. A question to ask is: what are the boundary conditions of POQ, or even further, of relative deprivation? As emphasized by Liu and Mo [28], a lack of performance opportunity is the key to POQ’s negative consequences. We believe that perceived opportunities play an important role, though not necessarily as opportunities to demonstrate capabilities; rather, it depends on personal interests. For instance, some POQ employees may be more eager to earn salaries that match their qualifications. Nonetheless, the opportunities to improve one’s situation are crucial for individuals under relative deprivation. In the case of POQ college students, most of them are not able to transfer between colleges as employees hop between jobs. A realistic approach is to aim for opportunities after graduation. Fortunately, college is a place that offers such opportunities. As a result, POQ students are motivated to learn in college to prepare them for future opportunities. In the case of POQ employees, opportunities outside the company encourage them to search for better opportunities; as a result, POQ employees tend to leave their current organizations [26]. If a reasonable approach does not exist, people under relative deprivation may resort to protest or violence [20]. Additionally, if revolt is not practical, people may turn to drugs [21] or gambling [67] for comfort. Previous studies mainly focused on the negative consequences of POQ, while this study demonstrated the possibilities of positive results under the right conditions. Similarly, when an organization applies an outcome-based control strategy to evaluate salesmen’s performance, a positive relationship between POQ and work performance is observed [68].

In addition, this study contributes to existing research by connecting POQ with performance-approach goals. Being a big fish in a small pond creates inconsistencies between self-evaluation and social recognition. For POQ individuals, an inevitable question is, “If I am truly overqualified, how did I end up here?” This question may be challenged not only by other people but also by oneself. This may not be a problem for POQ employees because their qualifications are rather visible, such as their educational background. However, for college students, their academic potential is something that cannot be presented directly; instead, it requires them to demonstrate their potential during college learning. As a result, POQ students are motivated by performance-approach goals. In this way, they can maintain a positive self-evaluation that is in line with their previous self-image. Consistent with previous studies, this study also confirmed the positive effect of a performance-approach goal on learning engagement [69].

Comparing the results of this study with the results from extant undermatch studies, a question to ask is why undermatch was always associated with negative academic outcomes such as bachelor’s degree completion [14] and post-college outcomes [2], while, in contrast, Wang et al. [3] and this study found a positive relationship between POQ and learning motivation and engagement. In our opinion, there are three possible explanations. First, it might be due to the method of measurement. In undermatch studies, both undermatch and outcomes were measured with objective indicators; in contrast, they were measured by self-report questionnaires in POQ studies. Though subjective overqualification and objective overqualification showed some convergence on employees (*r* = 0.40) [1], they still have different predictive power for some outcomes, such as psychological well-being [16].

The second possible reason is a cultural difference. Most participants in the undermatch studies were from countries that are characterized as individualistic, such as the United States [2,14] and the Netherlands [5]. In contrast, participants in this study were from China, which is characterized as a collectivistic culture. Two meta-analyses demonstrated that such cultural difference is one of the important boundary conditions of POQ [1,60]: some negative effects of POQ are buffered in countries that are characterized as having a high-power distance or collectivistic culture. Therefore, the differences in outcomes could also be due to cultural differences.

The third, and the most possible reason in our opinion, is social difference. Economic disadvantages have been recognized as the main cause of undermatch [5,6], which occurs mainly because talented students cannot afford the tuition and living expenses of attending selective universities. In contrast, POQ in China is mainly caused by poor performance in the college entrance examination [3]. Tuition and living expenses would hardly be an obstacle because most top universities in China are state universities that charge less tuition and provide affordable dormitories, which can be fully covered by student loans [70]. As a result, for students in undermatched studies, their economic disadvantages would not only affect their enrollment but also have an enduring effect on their subsequent college learning and career decision-making. In contrast, poor performance on the entrance examination has less influence on subsequent college learning and later career choices. In addition, Chinese society is less tolerant of students who drop out of college. Indeed, there are few cases of people who succeed after dropping out of college in China, such as Bill Gates or Steven Jobs. A blessing in disguise, even for overqualified students, completing a bachelor’s degree is still a wise choice.

#### 6.1.2. The Relationship between POQ and Life Satisfaction

A very interesting finding of this study is the nonsignificant relationship between POQ and life satisfaction. This result is inconsistent with previous findings in two aspects. First, this finding is inconsistent with the study that found a negative relationship between POQ and life satisfaction [3]. We believe this can be explained by the parallel mediating mechanism found in this study. A major difference between these two studies is that our participants were college seniors who had at least one-year experience in colleges, while their participants were freshmen who had just entered college and had been there for less than one month. At the start of college learning, POQ students have yet to walk away from their failures on the entrance exam; meanwhile, the effect via generalized self-efficacy was weak because they did not have the chance to utilize their academic potential in college learning. For senior students, those two effects may be reversed as they stay in college. On the one hand, they should become less concerned about their failures on the entrance exam over time, and on the other hand, they can consistently build their self-efficacy with their surplus abilities through college learning.

Second, the relationship between POQ and generalized self-efficacy was not supported by previous POQ studies in the workplace [60]. Although POQ represents a kind of positive self-evaluation, POQ employees are not able to exercise their surplus KSAs in their daily work [64]. Since utilizing one’s abilities is an important source of self-efficacy in the workplace [28], it is not surprising that POQ does not contribute to employees’ self-efficacy. However, for POQ college students, college learning is a desired activity to utilize their surplus academic potential. When their academic potential is recognized and rewarded in college learning, they can consistently build self-efficacy from it. Integrating the relationship between POQ and learning motivation with the relationship between POQ and self-efficacy, we can even anticipate a reciprocal relationship between those two effects.

#### 6.1.3. The Moderating Role of a Growth Mindset

Another contribution of this study is that we provided additional evidence that a growth mindset is less beneficial for students who are already confident about their abilities. Consistent with previous studies that found a fixed mindset enhanced the performance of favorable stereotyped groups [41], this study found that a growth mindset weakens the motivations provided by POQ. In the eyes of POQ students with a growth mindset, their advantages are not permanent. In developing their abilities, the role of their colleges cannot be neglected. The differences in resources and opportunities between general and selective universities are something that cannot be made up by their talent. In contrast, in the eyes of POQ students with a fixed mindset, the selectivity of the university will not affect their inherent ability. As long as they acquire enough knowledge and skills, they will not lose in competitions with students from selective universities. As a result, students with a fixed mindset are more motivated by POQ.

Despite the moderating role of the growth mindset, it should be stressed that our findings did not contradict the growth mindset theory, which emphasizes its benefits [38]. In this study, students with a higher growth mindset tended to score higher on motivation variables, though the gap was narrowed among students who scored high on the POQ. In other words, the direct effect of having a growth mindset was stronger than its moderating effect. In our opinion, the result can be better interpreted as POQ complementing the negative effect of a fixed mindset to some extent.

### 6.2. Practical Implications

At first glance, POQ students seem difficult to deal with. They may complain a lot because they are unsatisfied with their colleges [3]. Previous undermatch studies also suggested that they may also have academic difficulties [6,14]. However, the findings of this study, although they require further replication and validation, imply that college students who perceive themselves to be overqualified are less problematic than we expected. In fact, we found that POQ students are highly motivated to learn in college. This kind of high motivation was observed not only for first-year students but also college seniors. Since performance opportunities are crucial for POQ individuals [28], we suggest that colleges can inspire their motivation by providing various opportunities for their growth. Additionally, it has been suggested that lack of recognition is the major reason why POQ leads to deviant behaviors [28]. Therefore, we suggest that college teachers build good relationships with overqualified students. In this way, overqualified college students will feel that they are recognized by their teachers, which will enhance their learning engagement [71].

In addition, the negative side of POQ seems to weaken as students stay in college. Although previous studies have found that POQ negatively predicted freshmen’s life satisfaction [3], two independent surveys in this study suggested that the effect was nonsignificant for college seniors. The mediating effect analysis suggested that such a change was largely due to an increase in generalized self-efficacy. Therefore, we suggest that college teachers encourage POQ students to participate in challenging activities that can help build self-efficacy, such as academic competitions. In this way, their dissatisfaction caused by relative deprivation can be overcome. College teachers and counselors can also take actions to improve the generalized self-efficacy of freshmen and minimize the negative effect of POQ on life satisfaction in the early college years. For example, some intervention plans that focus on improving first-year college students’ self-efficacy showed effective results [72]. Therefore, we suggest college teachers and counselors utilize these plans with overqualified students who have decreased life satisfaction.

The moderating role of the growth mindset should also be stressed. Although a growth mindset weakened the relationship between POQ and motivations, it does not mean that a fixed mindset is more beneficial for POQ students; students with growth mindsets still scored higher on motivation variables. A more meaningful question to ask is how a growth mindset weakens the positive effect of POQ. If interventions can help students with a growth mindset be motivated by POQ as much as students with a fixed mindset, their academic performance will be further improved. Despite that, this finding suggests that intervention with a growth mindset may be less effective for students who are already confident in their abilities, which is consistent with the findings of a meta-analysis [11]. Considering the fact that such interventions have been widely adopted [38], we suggest practitioners focus more on disadvantaged groups when adopting such interventions.

Finally, our findings provide insight into the self-adjustment of overqualified college students. Although attending an overqualified college is not an optimal choice for most students, our findings suggest that this journey is less disappointing than they may have expected. In a less competitive academic environment, overqualified students can still utilize and develop their abilities and talents on campus. The key to self-adjustment is to evolve in learning activities, in which overqualified students can build their self-efficacy and improve life satisfaction. Therefore, we want to encourage overqualified college students to actively engage in college learning in order to cope with their sense of overqualification.

### 6.3. Limitations and Future Studies

The major limitation of this study is the cross-sectional design, from which causality cannot be guaranteed. There is a chance that students who possess high levels of learning motivation and self-efficacy tend to perceive themselves as overqualified. A longitudinal study design would help clarify this issue. Second, participants in this study were all from China; the homogeneity of participants should be acknowledged when interpreting our findings. Third, all variables were measured with self-reported questionnaires. A common method bias may affect the results. Future studies can consider using others’ evaluations as a source of data. For future studies, the trajectory of the relationship between college students’ POQ and life satisfaction as students’ stay in college remains unclear. We suggest future studies stress the issue and find the turning point, as well as factors that contribute to this reversal. In addition, we believe cross-cultural studies are also necessary and valuable. To our knowledge, existing college students’ POQ studies only focus on Chinese students. Since previous meta-analyses suggested that cultural factors moderate the relationship between POQ and outcomes in the workplace [1,60], a similar effect may also exist for college students.

## Figures and Tables

**Table 1:** Results of measurement invariance test.

Models	χ^2^ (df)	CFI	RMSEA	Model Compared	Δχ^2^ (df)	ΔCFI	ΔRMSEA
M1: Configural model	1134.790 (492)	0.899	0.079				
M2: Metric invariance	1181.357 (524)	0.897	0.077	M1	46.568 (32) *p* = 0.046	0.002	0.002
M3: Scalar invariance	1227.318 (556)	0.894	0.076	M2	45.961 (32) *p* = 0.052	0.002	0.001
M4: Residual invariance	1273.528 (596)	0.893	0.073	M3	46.228 (40) *p* = 0.231	0.001	0.002

**Table 2:** Means, standard deviations, and correlations of study 1.

Variables	M	SD	1	2	3	4	5	6	7	8
1. Perceived overqualification	3.15	0.54	-							
2. Achievement aspiration	3.69	0.51	0.22 **	-						
3. Educational aspiration	3.76	0.55	0.25 **	0.60 **	-					
4. Performance-approach goal	3.44	0.74	0.29 **	0.46 **	0.33 **	-				
5. Performance-avoidance goal	3.45	0.79	0.02	0.08 *	0.05	0.29 **	-			
6. Learning engagement	3.58	0.55	0.29 **	0.42 **	0.47 **	0.35 **	−0.02	-		
7. Growth mindset	2.66	0.23	−0.04	0.34 **	0.32 **	0.08 **	−0.16 **	0.35 **	-	
8. Relative deprivation	2.58	0.84	0.24 **	0.08 *	0.01	0.21 **	0.23 **	−0.04	−0.24 **	
9. Life satisfaction	4.01	1.00	0.08	0.02	0.11 **	0.08 **	0.03	0.38 **	0.16 **	−0.10 **

* *p* < 0.05, ** *p* < 0.01.

**Table 3:** Direct effect analysis results of POQ on learning engagement.

Outcomes	Predictors	*β*	SE	*p*	F	R^2^
Career aspiration	Constant	2.96	0.12	<0.01	13.90	0.08
	Perceived overqualification	0.25	0.03	<0.01		
	Gender	−0.07	0.04	0.06		
	Grade	0.00	0.02	0.93		
	Family monthly income	0.00	0.00	0.34		
Performance-approach goal	Constant	2.35	0.19	<0.01	16.67	0.09
	Perceived overqualification	0.41	0.05	<0.01		
	Gender	−0.04	0.06	0.53		
	Grade	−0.05	0.03	0.11		
	Family monthly income	0.00	0.00	0.05		
Performance-avoidance goal	Constant	3.47	0.21	<0.01	0.72	0.00
	Perceived overqualification	0.02	0.06	0.76		
	Gender	0.05	0.07	0.42		
	Grade	−0.03	0.04	0.46		
	Family monthly income	0.00	0.00	0.21		
Learning engagement	Constant	2.58	0.14	<0.01	17.55	0.10
	Perceived overqualification	0.32	0.04	<0.01		
	Gender	−0.10	0.04	0.02		
	Grade	0.01	0.02	0.61		
	Family monthly income	0.00	0.00	0.25		
Learning engagement	Constant	1.40	0.19	<0.01	33.54	0.26
	Perceived overqualification	0.17	0.04	<0.01		
	Career aspiration	0.38	0.04	<0.01		
	Performance-approach goal	0.13	0.03	<0.01		
	Performance-avoidance goal	−0.07	0.02	<0.01		
	Gender	−0.06	0.04	0.11		
	Grade	0.02	0.02	0.44		
	Family monthly income	0.00	0.00	0.49		

**Table 4:** Mediation effect analysis results of POQ on learning engagement.

Paths	Effect	SE	95% Confidence Interval
Total effect on learning engagement	0.32	0.04	[0.25, 0.40]
Direct effect	0.17	0.04	[0.10, 0.25]
Indirect effect via career aspiration	0.10	0.02	[0.06, 0.15]
Indirect effect via performance-approach goal	0.05	0.02	[0.02, 0.09]
Indirect effect via performance-avoidance goal	−0.00	0.01	[−0.01, 0.01]

**Table 5:** Moderating effect analysis results of growth mindset as a moderator.

Outcomes	Predictors	*β*	SE	*p*	F	R^2^
Career aspiration	Constant	3.73	0.06	<0.01	25.76	0.19
	POQ	0.25	0.03	<0.01		
	Growth mindset	0.70	0.07	<0.01		
	POQ × Growth mindset	−0.32	0.13	0.01		
	Gender	−0.05	0.04	0.19		
	Grade	0.01	0.02	0.58		
	Family monthly income	0.00	0.00	0.46		
Performance-approach goal	Constant	3.61	0.10	<0.01	12.94	0.11
	POQ	0.40	0.05	<0.01		
	Growth mindset	0.30	0.12	0.01		
	POQ × Growth mindset	−0.48	0.22	0.03		
	Gender	−0.02	0.06	0.75		
	Grade	−0.04	0.03	0.16		
	Family monthly income	0.00	0.00	0.05		
Learning engagement	Constant	2.19	0.17	<0.01	40.91	0.33
	POQ	0.20	0.04	<0.01		
	Career aspiration	0.25	0.04	<0.01		
	Performance-approach goal	0.12	0.03	<0.01		
	Growth mindset	0.69	0.08	<0.01		
	POQ × Growth mindset	−0.52	0.14	<0.01		
	Gender	−0.04	0.04	0.23		
	Grade	0.03	0.02	0.17		
	Family monthly income	0.00	0.00	−0.50		

**Table 6:** Moderated mediation effect analysis results of POQ on life satisfaction.

Path	Effect	SE	95% Confidence Interval
Direct effect			
Low growth mindset	0.32	0.05	[0.23, 0.41]
High growth mindset	0.08	0.05	[−0.01, 0.18]
Indirect effect via career aspiration			
Low growth mindset	0.08	0.03	[0.04, 0.14]
High growth mindset	0.04	0.02	[0.02, 0.08]
Indirect effect via performance-approach goal			
Low growth mindset	0.06	0.02	[0.02, 0.10]
High growth mindset	0.03	0.01	[0.01, 0.06]

**Table 7:** Direct effect analysis results of POQ on life satisfaction (study 1).

Outcomes	Predictors	*β*	SE	*p*	F	R^2^
Relative deprivation	Constant	1.36	0.21	<0.01	12.04	0.07
	POQ	0.34	0.06	<0.01		
	Gender	0.18	0.07	<0.01		
	Grade	0.04	0.04	0.27		
	Family monthly income	0.01	0.01	0.27		
Life satisfaction	Constant	3.55	0.27	<0.01	1.38	0.0′
	POQ	0.11	0.08	0.13		
	Gender	0.10	0.08	0.22		
	Grade	0.02	0.04	0.69		
	Family monthly income	0.00	0.00	0.61		
Life satisfaction	Constant	3.76	0.28	<0.01	3.35	0.01
	POQ	0.17	0.08	0.03		
	Relative deprivation	−0.16	0.05	<0.01		
	Gender	0.13	0.08	0.11		
	Grade	0.02	0.04	0.58		
	Family monthly income	0.00	0.00	0.72		

**Table 8:** Means, standard deviations, and correlations of study 2.

Variables	*α*	M	SD	1	2	3
1. Perceived overqualification	0.89	3.16	0.54	-		
2. Relative deprivation	0.69	2.53	0.78	0.38 **	-	
3. Generalized self-efficacy	0.74	4.22	0.65	0.33 **	−0.03	-
4. Life satisfaction	0.87	3.86	1.00	0.16	−0.20 *	0.38 **

* *p* < 0.05, ** *p* < 0.01.

**Table 9:** Direct effect analysis results of POQ on life satisfaction (study 2).

Outcomes	Predictors	*β*	SE	*p*	F	R^2^
Relative deprivation	Constant	1.03	0.46	0.03	8.28	0.22
	POQ	0.54	0.12	<0.01		
	Gender	0.50	0.16	<0.01		
	Grade	−0.07	0.09	0.41		
	SSES	−0.03	0.05	0.62		
Generalized self-efficacy	Constant	3.10	0.40	<0.01	4.41	0.13
	POQ	0.40	0.10	<0.01		
	Gender	−0.11	0.14	−0.81		
	Grade	−0.10	0.08	0.20		
	SSES	0.03	0.04	0.56		
Life satisfaction	Constant	2.48	0.63	<0.01	3.63	0.11
	POQ	0.26	0.16	0.10		
	Gender	−0.23	0.22	0.29		
	Grade	−0.11	0.12	0.35		
	SSES	0.19	0.07	<0.01		
Life satisfaction	Constant	1.39	0.73	0.06	6.51	0.25
	POQ	0.25	0.17	0.16		
	Relative deprivation	−0.31	0.12	<0.01		
	Generalized self-efficacy	0.46	0.13	<0.01		
	Gender	−0.03	0.21	0.90		
	Grade	−0.08	0.11	0.43		
	SSES	0.17	0.06	<0.01		

**Table 10:** Mediation effect analysis results of POQ on life satisfaction.

Paths	Effect	SE	95% Confidence Interval
Total effect	0.26	0.16	[−0.05, 0.58]
Direct effect	0.25	0.17	[−0.10, 0.59]
Indirect effect via relative deprivation	−0.17	0.08	[−0.34, −0.03]
Indirect effect via generalized self-efficacy	0.18	0.09	[0.04, 0.40]

## Data Availability

Data is available upon reasonable request.
